# Acral Mesenchymal Spindle Cell Neoplasm With a Novel *HMGA2::NCOA2* Fusion

**DOI:** 10.1111/cup.70041

**Published:** 2025-12-29

**Authors:** Grace Z. Armstrong, Carina A. Dehner, Eitan Halper‐Stromberg, Esther Baranov, Anna C. Eden, Rachel P. Kowal

**Affiliations:** ^1^ Department of Pathology and Laboratory Medicine Indiana University School of Medicine Indianapolis Indiana USA; ^2^ Department of Pathology and Laboratory Medicine University of Pennsylvania Philadelphia Pennsylvania USA; ^3^ Department of Dermatology Indiana University School of Medicine Indianapolis Indiana USA

**Keywords:** HMGA2, mesenchymal tumor, NCOA2

## Abstract

Molecular profiling has revolutionized the field of soft tissue pathology, enhancing diagnostic precision and treatment strategies. The integration of molecular analysis and immunohistochemistry has been crucial for classifying diagnostically challenging acral mesenchymal neoplasms. Herein, we report the first documented case of an acral mesenchymal spindle cell neoplasm harboring an *HMGA2::NCOA2* fusion. The neoplasm presented as a slow‐growing verrucous papule on the right thumb of an 18‐year‐old female. Histological examination revealed spindled cells of varying cellularity with intervening sclerotic collagen and dilated vasculature. The cells had patchy S100 and focal GLUT‐1 reactivity but were negative for CD34, EMA, Sox‐10, Pan‐TRK, p63, CKAE1/3, MUC4, ALK, Factor 13A, actin, desmin, and ERG. Given the unusual morphology and non‐diagnostic immunohistochemical profile, the specimen was sent for additional molecular profiling. Next‐generation sequencing revealed a novel in‐frame *HMGA2*::*NCOA2* fusion. The tumor was likely benign, with 4 mitoses per 10 high‐powered fields (HPF), but was excised due to the unpredictable behavior of the fusion. As the first known case to date with this fusion, these findings contribute to the emerging research on genomic testing in acral soft tissue tumors. Additional cases and longer clinical follow‐up are needed to better characterize the novel *HMGA2::NCOA2* fusion.

## 1 Introduction

Acral mesenchymal neoplasms are a diverse group of soft tissue tumors that manifest on the distal extremities. They are difficult to diagnose due to overlapping morphology and often require the integration of immunohistochemistry and molecular analysis for classification. Recent advancements in molecular profiling have transformed the field of soft tissue pathology, improving diagnostic accuracy and medical management. The identification of fusion‐associated entities has expanded our knowledge of tumor pathogenesis and continues to be a growing field of research. In this study, we report an acral mesenchymal spindle cell neoplasm harboring a novel *HMGA2::NCOA2* fusion. Since this is the first known case to date, we also discuss potential differential diagnoses and clinical recommendations.

## 2 Case Report

**FIGURE 1:**
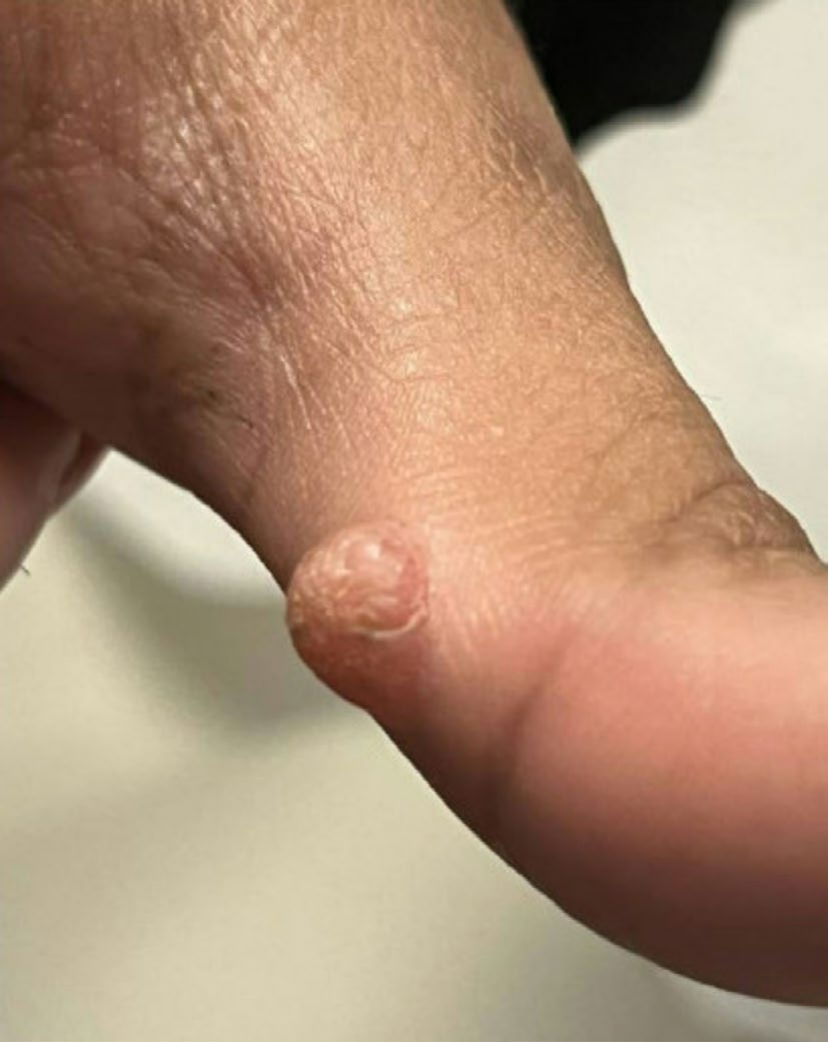
Clinical photograph of the right thumb lesion.

An 18‐year‐old female presented with a 1 cm exophytic, verrucous papule on the right palmar base of the thumb (Figure 1). The lesion was painless and slowly growing over the course of a year. A shave biopsy was performed 1 month later, revealing a spindle cell proliferation of indeterminate significance.

**FIGURE 2:**
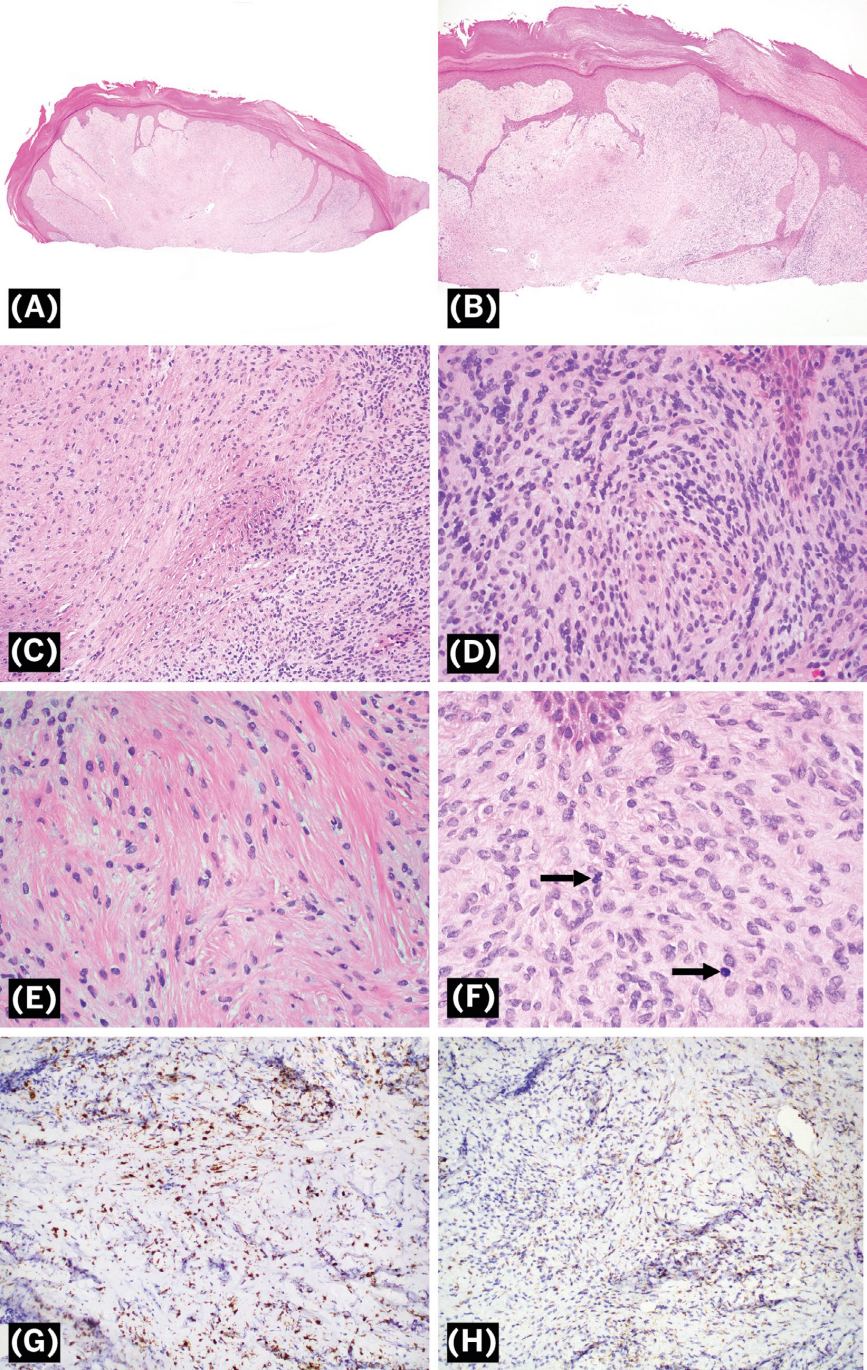
Morphologic features of an acral mesenchymal spindle cell neoplasm with a novel *HMGA2::NCOA2* fusion. (A) Low‐power view of dermal spindle cell proliferation with dilated vasculature (H&E, 2×). (B) Acanthotic epidermis with connections into the deep margin (H&E, 4×). (C) Varying degrees of cellularity with hypocellular and hypercellular areas of relatively bland spindle cells (H&E, 20×). (D) High‐power view of hypercellular area (H&E, 40×). (E) High‐power view of hypocellular area with intervening sclerotic collagen (H&E, 40×). (F) Arrows pointing to scattered mitotic figures (H&E, 60×). (G) S100 shows patchy reactivity (20×). (H) GLUT‐1 shows focal reactivity (20×).

Histopathological sections showed a dermal proliferation of spindled cells abutting the epidermis and extending to at least the mid‐dermis and diffusely to the deep margin. The epidermis was acanthotic and hyperplastic with anastomosing elongated connections that extended to the deep margin. The spindled cells exhibited varying degrees of cellularity throughout the dermis with less cellular areas admixed with sclerotic collagen and superficial dilated vascular channels (Figure 2A–E). The cells appeared relatively bland with indistinct cell borders and inconspicuous nucleoli. No giant cells were identified. Scattered superficial mitotic figures were identified with 4 mitoses per 10 high‐powered fields (HPF) (Figure 2F). Immunohistochemical stains for CD34, EMA, Sox‐10, Pan‐TRK, p63, CKAE1/3, MUC4, ALK, Factor 13A, actin, desmin, and ERG were non‐reactive in lesional cells. There was patchy reactivity for S100 and very focal staining with GLUT‐1 (Figure 2G,H).

**FIGURE 3:**
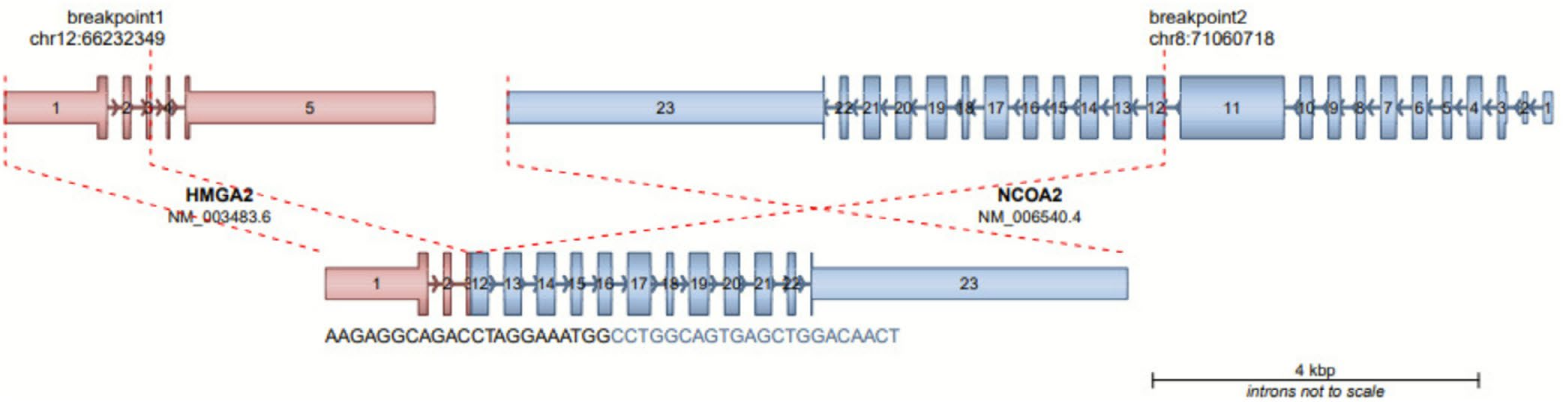
Next‐generation sequencing identified an *HMGA2::NCOA2* fusion. The in‐frame fusion mapped to *HMGA2* exon 3 and *NCOA2* exon 12.

Due to the unusual morphology and non‐diagnostic immunohistochemical profile, the specimen was sent to Tempus AI Inc. for additional molecular profiling. Next‐generation sequencing revealed a novel *HMGA2* exon 3::*NCOA2* exon 12 in‐frame fusion (Figure 3). Given the lack of specific morphologic or immunohistochemical features and no previously reported cases with this fusion at the time of this report, definitive classification remains challenging. Although no overt features of malignancy were seen, excision was recommended due to the unpredictable behavior of the novel fusion. The lesion was excised 3 months later and will be monitored for recurrence.

## 3 Discussion

Herein, we report the first known case to date of an acral spindle cell neoplasm with an *HMGA2::NCOA2* fusion. The novel tumor was composed of spindled cells surrounded by dilated vascular channels and sclerotic collagen. Immunohistochemistry demonstrated patchy S100 and focal GLUT‐1 staining but no reactivity for CD34, EMA, Sox‐10, Pan‐TRK, p63, CKAE1/3, MUC4, ALK, Factor 13A, actin, desmin, and ERG.

*HMGA2*, also known as high mobility group AT‐hook 2, is on chromosome 12q14.3 and encodes a protein in the non‐histone chromosomal high mobility group (HMG) protein family [1]. This protein acts as a transcription factor that increases cancer proliferation by inhibiting apoptosis and promoting cell cycle entry [2]. *HMGA2* gene fusions have been reported in a variety of mesenchymal tumors, including adipocytic tumor (*HMGA2::KERA* and *HMGA2::SETBP1*) [3, 4], chondroid hamartoma (*HMGA2::LPP*) [5], chondroma (*HMGA2::LPP*) [6], keratin‐positive giant cell tumor (*HMGA2::NCOR2*) [7], leiomyosarcoma (*HMGA2::RAD51B*) [8], lipoblastoma (*HMGA2::GSN*) [9], liposarcoma (*HMGA2::KITLG*) [10], osteochondroma (*HMGA2::SOX5*) [11], rhabdomyosarcoma (*RAB3IP::HMGA2*) [12], as well as multiple leiomyomas, lipomas, and malignant melanomas.

The fusion counterpart *NCOA2* (nuclear receptor coactivator 2) is located on chromosome 8q13.3 [13]. The gene encodes a transcriptional coactivator for nuclear hormone receptors, including steroid, retinoid, and thyroid receptors [13]. Fusions involving the *NCOA2* gene have been indicated in numerous mesenchymal tumors, such as angiofibroma (*AHRR::NCOA2* and *NCOA2::ETV4*) [14, 15], chondrosarcoma (*HEY1::NCOA2*) [16], myxoid epithelioid smooth muscle tumor (*MEF2D::NCOA2*) [17], malignant melanoma (*NCOA2::ST18*) [18], and different subtypes of rhabdomyosarcomas [19, 20].

The diagnosis of our acral mesenchymal spindle cell neoplasm is challenging, as there are multiple morphologic differentials with similar histologic and immunohistochemical features. It may be confused with *HMGA2::NCOR2* fusions found in keratin‐positive giant cell‐rich tumors (KPGCTs); however, our neoplasm is differentiated morphologically by the absence of multinucleated giant cells and keratin reactivity [21].

Given the spindle cell morphology and location, acral fibromyxoma was considered. This neoplasm, also known as superficial acral fibromyxoma (SAFM) or digital fibromyxoma, typically occurs in subungual or periungual areas and is characterized by spindled to stellate cells organized in a loose fascicular pattern surrounded by dense hyaline collagen and myxoid stroma [22]. While these neoplasms have a similar morphology to our case, they most often demonstrate diffuse CD34 expression and loss of Rb1 on immunohistochemistry [23].

Sclerosing perineurioma is another acral soft tissue tumor that predominantly forms on the hands [24, 25]. However, these rare tumors exhibit different morphologic features including collagen bundles with a whorled onion bulb‐like pattern of epithelioid and spindled cells [25]. Furthermore, this tumor is reactive for EMA [24], which was not seen in our case.

Acral fibrochondromyxoid tumor (AFCMT) is a recently described neoplasm affecting the distal extremities harboring a unique *THBS1::ADGRF5* gene fusion [26]. Histologically, these tumors are composed of clusters of chondrocyte‐like cells in a chondromyxoid stroma surrounded by vascular septa [27] in contrast to the predominantly spindled morphology and the fibrous stroma seen in our case. Furthermore, AFCMTs have ERG and CD34 reactivity [26, 27].

Another emerging fusion‐defined soft tissue tumor with an acral predilection is the *SMAD3*‐rearranged fibroblastic tumor [28, 29]. Histologic examination demonstrates hypercellular fascicles of uniform spindled cells and prominent hypocellular areas of hyalinization with focal calcifications [29]. *EWSR1::SMAD3* positive fibroblastic tumors are consistently positive for ERG and negative for other markers [28].

An additional acral mesenchymal tumor with a characteristic gene fusion is the hyalinizing epithelioid tumor with *OGT::FOXO* fusion [30, 31]. This rare neoplasm has a distinctive morphology characterized by nests of epithelioid cells in a hyalinized and myxoid stroma [30]. Hyalinizing epithelioid tumors can also be distinguished by focal EMA and CD34 reactivity [31].

This report presents an acral mesenchymal spindle cell proliferation of the hand with a novel *HMGA2::NCOA2* fusion. Since this is the first reported case of *HMGA2::NCOA2* in an acral mesenchymal neoplasm, the clinical behavior of this tumor is unclear and clinical monitoring to ensure against local recurrence was recommended. Further studies are required to determine the molecular and clinical significance of this fusion.

## Author Contributions

Grace Z. Armstrong, Rachel P. Kowal, and Carina A. Dehner conceptualized the study, acquired the data, drafted the manuscript, and provided critical revisions. Eitan Halper‐Stromberg, Esther Baranov, and Anna C. Eden contributed to data collection and manuscript editing. All authors approved the final manuscript.

## Ethics Statement

In accordance with institutional guidelines, ethical approval was not required for this case report. Efforts were made to fully anonymize the case details to safeguard the patient's privacy.

## Conflicts of Interest

The authors declare no conflicts of interest.

## Data Availability

The data that support the findings of this study are available on request from the corresponding author. The data are not publicly available due to privacy or ethical restrictions.
